# Navigational choice between reversal and curve during acidic pH avoidance behavior in *Caenorhabditis elegans*

**DOI:** 10.1186/s12868-015-0220-0

**Published:** 2015-11-19

**Authors:** Tokumitsu Wakabayashi, Kazumi Sakata, Takuya Togashi, Hiroaki Itoi, Sayaka Shinohe, Miwa Watanabe, Ryuzo Shingai

**Affiliations:** Department of Chemistry and Biological Sciences, Faculty of Engineering, Iwate University, Iwate, 020-8551 Japan

**Keywords:** *Caenorhabditis elegans*, Acidic pH avoidance, Navigation, Sensory**-**motor transformation

## Abstract

**Background:**

Under experimental conditions, virtually all behaviors of *Caenorhabditis elegans* are achieved by combinations of simple locomotion, including forward, reversal movement, turning by deep body bending, and gradual shallow turning. To study how worms regulate these locomotion in response to sensory information, acidic pH avoidance behavior was analyzed by using worm tracking system.

**Results:**

In the acidic pH avoidance, we characterized two types of behavioral maneuvers that have similar behavioral sequences in chemotaxis and thermotaxis. A stereotypic reversal-turn-forward sequence of reversal avoidance caused an abrupt random reorientation, and a shallow gradual turn in curve avoidance caused non-random reorientation in a less acidic direction to avoid the acidic pH. Our results suggest that these two maneuvers were each triggered by a distinct threshold pH. A simulation study using the two-distinct-threshold model reproduced the avoidance behavior of the real worm, supporting the presence of the threshold. Threshold pH for both reversal and curve avoidance was altered in mutants with reduced or enhanced glutamatergic signaling from acid-sensing neurons.

**Conclusions:**

*C. elegans* employ two behavioral maneuvers, reversal (klinokinesis) and curve (klinotaxis) to avoid acidic pH. Unlike the chemotaxis in *C. elegans*, reversal and curve avoidances were triggered by absolute pH rather than temporal derivative of stimulus concentration in this behavior. The pH threshold is different between reversal and curve avoidance. Mutant studies suggested that the difference results from a differential amount of glutamate released from ASH and ASK chemosensory neurons.

**Electronic supplementary material:**

The online version of this article (doi:10.1186/s12868-015-0220-0) contains supplementary material, which is available to authorized users.

## Background

Neuronal circuits transform sensory inputs into behavior in animals. Elucidating the mechanism of the sensory-motor transformation pathway is one of important issues in neurobiology. In *Caenorhabditis elegans*, practically all behavior under experimental conditions are achieved through sets of simple locomotion, including forward and backward movement, turning by deep body bending, shallow gradual reorientations, and resting [1, 2]. Worms adaptively combine these types of locomotion according to sensory cues. Therefore, the initial step in understanding the mechanism of the sensory-motor transformation pathway in *C. elegans* is to determine how the worms regulate their locomotion in response to environmental stimuli.

*C. elegans* shows chemotaxis and thermotaxis toward favorable chemicals and temperature, respectively [3, 4], both of which are achieved through three behavioral strategies: (1) biased random walk (i.e., klinokinesis), a repeated, abrupt, random reorientation until the worm is headed in a favorable direction [5–7], (2) non-random steering (i.e., klinotaxis), a gradual reorientation in a favorable direction [8, 9], and (3) slowing response (i.e., orthotaxis), deceleration allowing the worm to remain in favorable conditions [10]. By the analyses of locomotion of the worms, in which specific neurons or genes were inactivated, combined with defined stimulus application in temporal and spatial manner, function of sensory neurons and the downstream neuronal circuit involved in attraction behaviors were studied in detail [7, 9, 11–16]. However, analysis of locomotion during avoidance behavior to a spatial gradient of repellents is relatively limited to date [17–19].

In this study, locomotion of *C. elegans* during acidic pH avoidance behavior was analyzed. Similar to the attraction behavior, behavioral maneuvers probably corresponding to biased random walk and non-random steering were observed in the avoidance. Apparently, these behavioral maneuvers were triggered by the threshold pH for each maneuver, rather than by temporal variation in the repellent concentration. The apparent threshold pH was regulated, at least in part, by glutamatergic neurotransmission by ASH and ASK neurons.

## Results

### Behavioral maneuvers observed during acidic pH avoidance

**Fig. 1:**
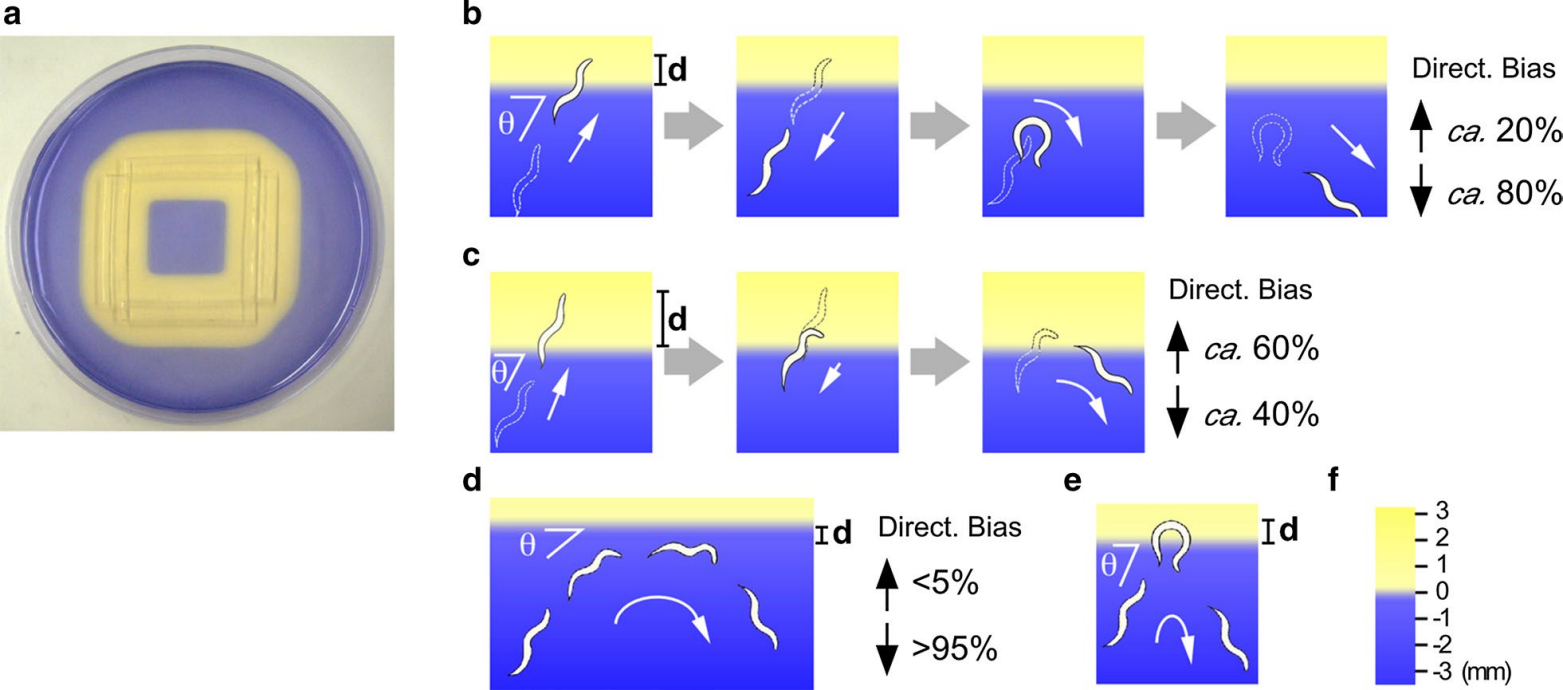
Assay plate and acidic pH avoidance behavior. **a** Assay plate used in this study. Because of the pH-sensitive BPB dye added to the assay plate, the less acidic region became blue and the acidic region (approximately pH 4.0) turned yellow (see “Methods” for detail). Schematic drawings of **b** long reversal avoidance, **c** short reversal avoidance, **d** gradual curve avoidance, and **e** deep curve avoidance behavior. The angle of encounter into the acidic region (theta), the distance of worm position relative to the acidic yellow color border (**d**), and directional bias after each behavioral maneuver are indicated. Because deep curve avoidance was rarely observed, directional bias was not indicated. **f** Positive and negative directions of distance relative to the color border used in the following figures. *Scale* in **f** is not identical to that in (**b**–**e**)

To explore the locomotory behavior of *C. elegans* on a spatial gradient of repellent, we used acidic pH as a repulsive sensory stimulus. *C. elegans* has been shown to avoid acidic pH less than 4.0 [20]. Unlike other water-soluble chemoattractants and repellents, the concentration gradient of protons (i.e., acidic pH gradient) can be visualized by adding pH-sensitive dye to the assay plate (Fig. 1a). Since the acidic yellow border of BPB dye (around pH 4.0) closely corresponds to the acidic pH avoided by worms, we can record worm avoidance behavior along with the direction and position of the worm relative to the color border.

In our observation, *C. elegans* showed four types of behavior during acidic pH avoidance. In long reversal avoidance (Fig. 1b, Additional file 1: Movie S1), an abrupt long backward locomotion of three or more body undulations followed by reorientation by deep body bending and subsequent forward locomotion in a less acidic direction were observed when the worm came closer to the acidic color border. Gray et al. [21] described that the initial deep body bending (omega turn) immediately after long reversal cause a reorientation having an average angle of 137° ± 8° toward ventral side. Because of this deep body bending, the direction of the worm locomotion after long reversal avoidance was biased in a less acidic direction. In short reversal avoidance (Fig. 1c), an abrupt short backward locomotion of two or less body undulations, followed by forward locomotion, were observed near the acidic yellow border. During this behavior, directional change after the short backward locomotion was always small (less than 45°), and reorientation bias in a less acidic direction was not observed. When the worm failed to reorient itself in a less acidic direction after the short reversal avoidance, it repeated the avoidance behavior until it could escape from the acidic region, similar to the biased random walk strategy in worm chemotaxis [5]. In the present study, we collectively referred to these two types of behavior as reversal avoidance. The behavioral parameters described below were indistinguishable between long and short reversal. In the gradual curve avoidance (Fig. 1d, Additional file 2: Movie S2), worms avoiding the acidic region kept moving forward with gradual reorientations. The deep curve avoidance (also known as omega turn, Fig. 1e), in which worms bend their body deeply in a less acidic direction, was rarely observed. We collectively referred to these two types of behavior as curve avoidance. In successful curve avoidance, the direction of forward movement after the behavioral maneuver was biased in a less acidic direction, suggesting non-random reorientation during the acidic pH avoidance behavior.

### Navigational choice during acidic pH avoidance behavior is dependent on the bearing

**Fig. 2:**
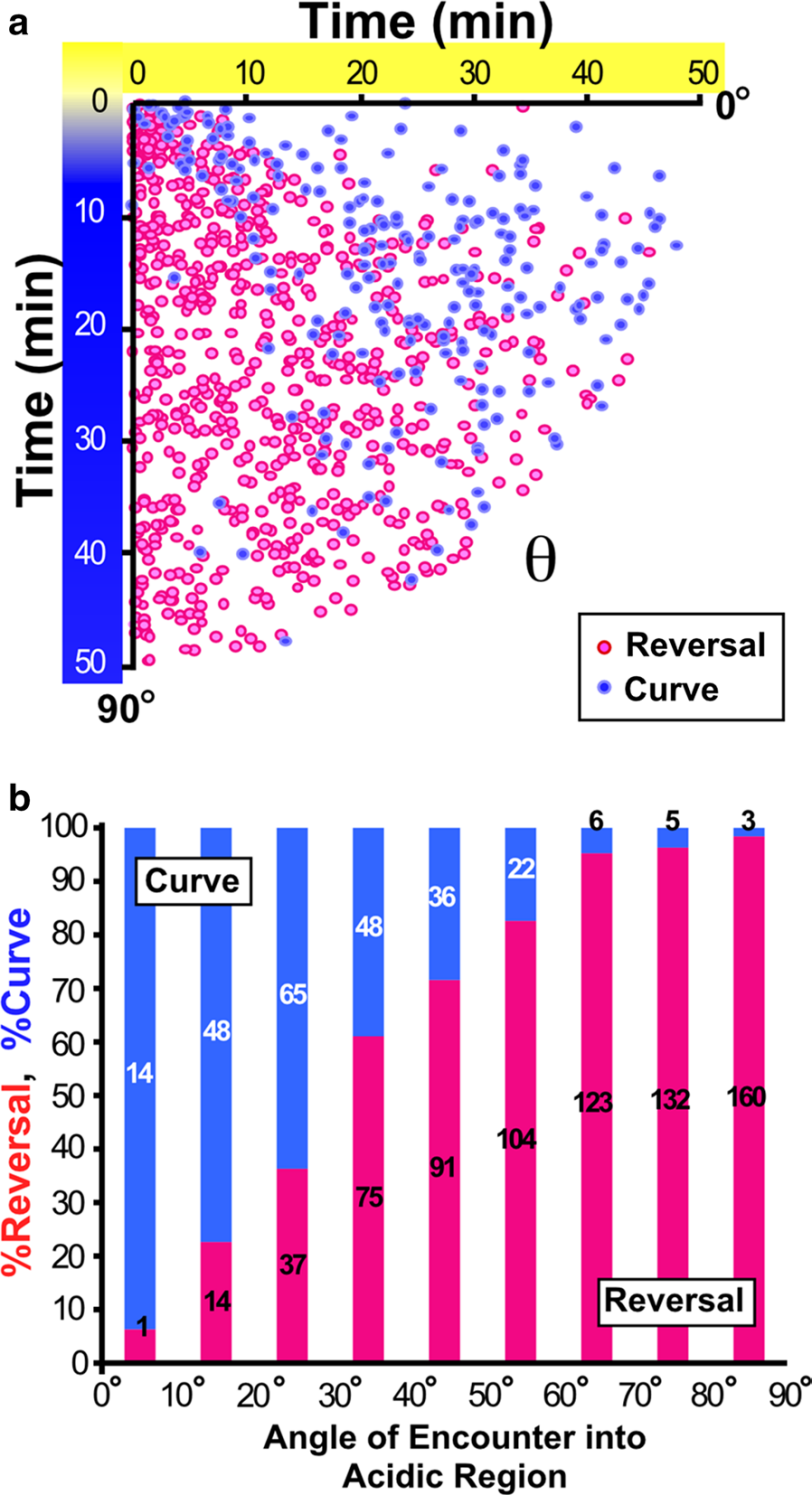
Angle dependent choice of reversal and curve avoidance. **a** Relationship between the angle of encounter and behavioral maneuvers chosen during acidic pH avoidance behavior in wild-type *C. elegans*. The distance from the origin of the coordinate to each *dot* represents the time at which the behavior occurred. The angle between the horizontal axis and a line between the origin and each dot represents the angle of encounter into the acidic region (theta in Fig. 1). We only considered the acute angle in every avoidance behavior. **b** The ratio of the behavioral choice between curve and reversal avoidance, binned every 10 degrees. The number of events observed in each bin has been indicated in the bar graph. In most experiments, we observed around 500 avoidance events from more than 10 worms. Acidic agar strips containing 100 mM HCl were used to form a gradient on a plate containing 5 mM potassium phosphate buffer (pH 6.0)

In this study, we analyzed two parameters: (1) the angle between the direction of forward locomotion prior to reorientation and the acidic color border (angle of encounter), and (2) the position of the worm relative to the acidic color border when the worm entered furthest into (or most closely approached) the acidic yellow region during the reorientation maneuver (Fig. 1b–e). Figure 2a shows the relationship between the angle of encounter and the two reorientation maneuvers during the avoidance behavior. Behavioral choices between reversal and curve avoidance appeared to have been dependent on the angle of encounter (correlation coefficient: R = 0.92, p < 0.01) (Fig. 2a, b). In other words, the worms that encountered the acidic region at a deep, perpendicular angle approximating 90° chose reversal more often than curve. Conversely the worms that encountered the acidic region at a shallow angle chose curve more often than reversal. The ratio of reversal avoidance gradually increased and that of curve avoidance decreased with an increase in the angle of encounter (Fig. 2b). To see how the worms accomplish angle-dependent choice of reorientation maneuvers, we examined the avoidance behavior under various conditions, as described below.

### Effect of steepness of acidic pH gradient on navigational choice

Based on the previous observations on *C. elegans* chemotaxis behavior, it is widely accepted that the worm can sense temporal changes in attractant concentrations (*dC*/*dt*) caused by its own locomotion. Positive *dC*/*dt* suppresses reversal, and negative *dC*/*dt* promotes reversal [5, 15, 16]. In the acidic pH avoidance behavior, it is possible to think that the *dC*/*dt* of repellent is also involved in the angle-dependent navigational choice. When the worms encountered the acidic region at a deep angle, they sensed a larger *dC*/*dt* than when they encountered the acidic region at a shallow angle. This hypothesis implies that a large *dC*/*dt* causes reversal and a small *dC*/*dt* causes curve avoidance. In this scenario, the steepness of the acidic pH gradient should affect navigational choice. In a shallow gradient, *dC*/*dt* is consistently lower than in a steep gradient, thus the worm may chose more curve than in a steep gradient. In order to see the effect of the steepness of the gradient, we changed the buffer and made an acidic pH gradient using a lower concentration of HCl.

**Fig. 3:**
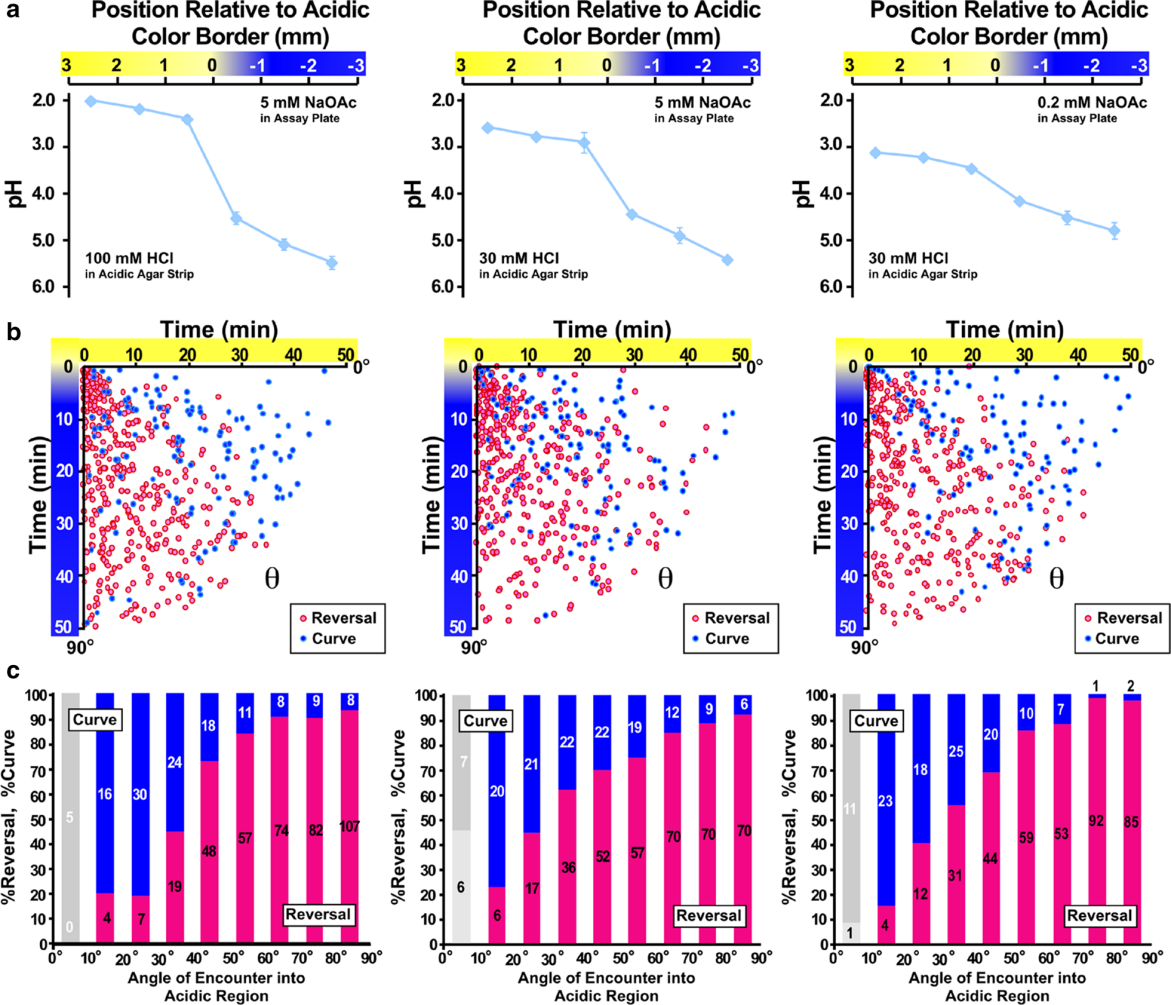
Acidic pH avoidance behavior of wild-type *C. elegans* on shallow acidic pH gradients. **a** The pH gradient measured from excised agar fragments from the assay plate. **b** Relationship between the angle of encounter and behavioral maneuvers. From *left* to *right*, results from relatively steep to shallow gradients depicted in (**a**) were shown. **c** The ratio of behavioral choice. Data were represented as in Fig. 2a, b. Results from 0 to 10 degrees bin were shaded in (**c**) because of the low number of avoidance events less than 15. The ratio of reversal had a positive correlation with the angle in all conditions (R > 0.9, *p* < 0.01)

The gradient became less steep when we used 100 mM, 30 mM HCl on 5 mM sodium acetate buffer (pH 6.0) and 30 mM HCl on 0.2 mM sodium acetate buffer (Fig. 3a). Under these conditions, however, the worms showed essentially the same pattern of angle dependence as in the steep gradient (Figs. 2, 3b, c). The ratio of curve avoidance did not increase even in the shallowest gradient examined. Therefore, *dC*/*dt* of the repellent (i.e., steepness of the gradient) may not be a major determinant of the angle-dependent navigational choice in this avoidance behavior.

### Two-distinct-threshold model on navigational choice during acidic pH avoidance

**Fig. 4:**
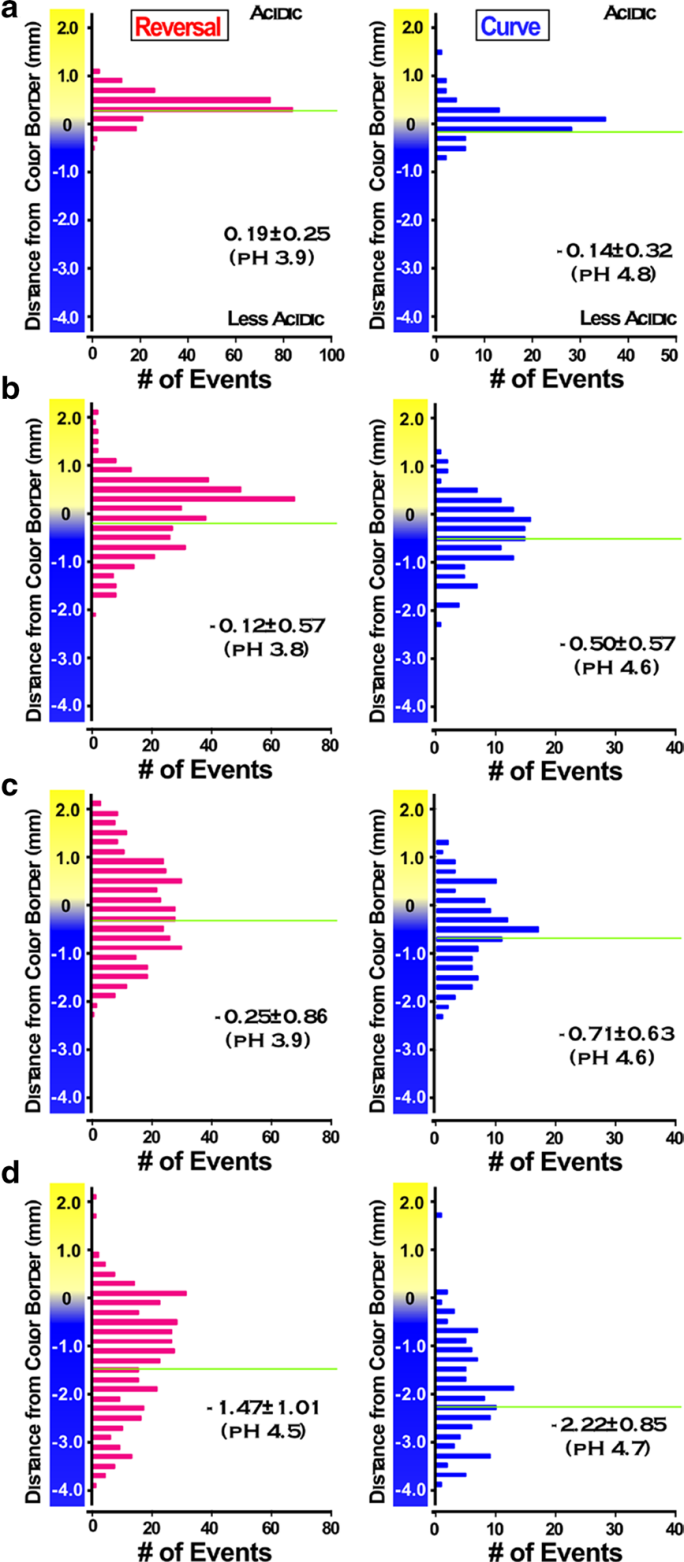
Histograms of the distance of the worm positions relative to the acidic yellow border. Histograms of the distance of the worm positions in reversal avoidance (*left*) and curve avoidance (*right*) binned every 0.2 mm. **a** Histograms of avoidance behavior obtained using 100 mM HCl to form gradients on a plate containing 5 mM potassium phosphate buffer (same condition as in Fig. 2, see Additional file 3: Fig. S4 for steepness). **b**–**d** Results obtained from **b** 100 mM HCl on 5 mM sodium acetate buffer, **c** 30 mM HCl on 5 mM sodium acetate buffer, and **d** 30 mM HCl on 0.2 mM sodium acetate buffer. *Horizontal lines* represent the mean distance. Mean ± SD and the pH at the position estimated from Fig. 3a were also indicated. Differences in variance were compared using the *F* test. Variance in **b**, **c**, and **d** were significantly different from that in **a** for both reversal and curve avoidance (*p* < 0.01)

In the course of analyzing worm position relative to the color border, we noted that under every condition examined, the mean distance at which the reversal avoidance occurred locates more acidic region than that at which curve avoidance occurred (Fig. 4). Although the difference between the mean distance for reversal and curve avoidance increased in shallower gradients, the pH values at the mean distance estimated from the measured pH gradient (Fig. 3a) were similar, especially for the curve avoidance. This observation led us to hypothesize that there are two distinct threshold pH values for reversal and curve. In this two-threshold model, the worm begins curving when it approaches the less acidic threshold pH for curve. If worm encounters the acidic region at a shallow angle, it can complete the curve avoidance with a single curve or multiple sequential curves without reaching the second, more acidic threshold pH for reversal. However, if the worm encounters the acidic region at a deep angle, it reaches the second threshold for reversal before it finishes its curve, thus executing reversal avoidance. Indeed, small reorientations like the beginning of curve avoidance were frequently observed immediately before reversal avoidance (Additional file 1: Movie S1). In addition, the worms showed small head swings (Additional file 3: Fig. S3), which were different from body undulation during the avoidance maneuvers. The small head swings may reflect the worm’s repeated sampling of the local pH to improve the fidelity of avoidance behavior, supporting the idea that the worm detects the local threshold pH rather than temporal variation of the stimuli caused by its locomotion. Taken together, these results suggest the presence of two distinct threshold pH values for the two avoidance maneuvers. These results also indicate that the apparent angle-dependent choice during acidic pH avoidance behavior is the result of the difference between these two threshold pH values.

The mean distance of the reversal and curve in Fig. 4 roughly corresponds to pH 3.8–4.0 and pH 4.6–4.8, respectively, when we estimated from the gradient measured in Fig. 3a. Each threshold pH may be found near or within the respective ranges. However, we could not estimate the precise threshold pH value because of the low spatial resolution of our pH measurements. Although the steepness of the repellent gradient did not affect the navigational choice, steepness of gradient did have marked effect on the variability of worm position at which avoidance was executed. Both curve and reversal avoidance occurred in a very narrow range of distance when a steep gradient was used, as opposed to that when shallow gradient was used (Fig. 4; Additional file 3: Fig. S4).

### Computer simulation of acidic pH avoidance using the two-distinct-threshold model

**Fig. 5:**
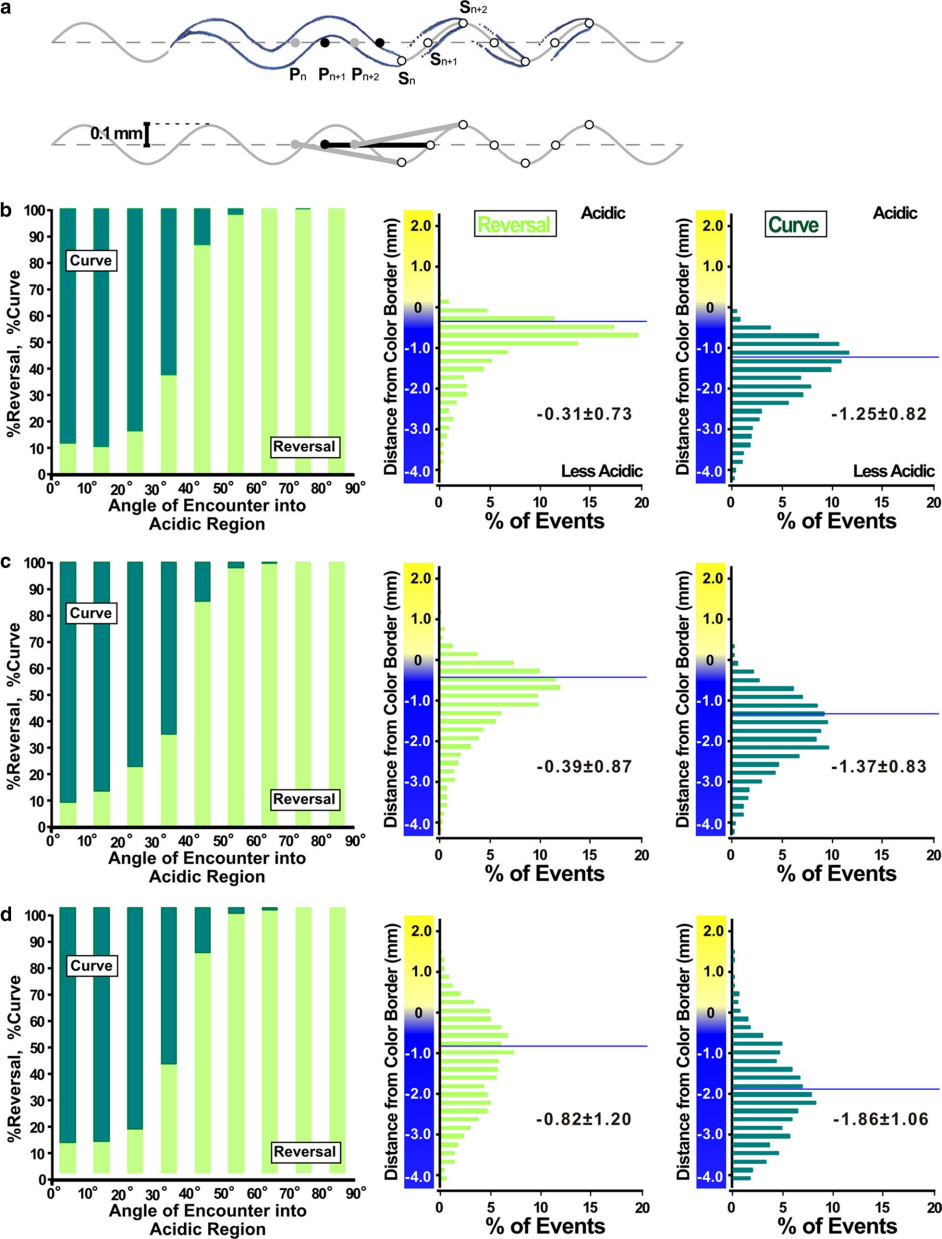
Computer simulation of acidic pH avoidance behavior using a two-distinct-threshold model. **a** Schematic drawings of the locomotion of the model worm. The position of the model worm (P_n_, P_n+1_,…) and the pH sensor of the worm (S_n_, S_n+1_,…) at each time point is shown by filled and open circles, respectively. **b**–**d** Behavior of the model worm. Results are shown as in Fig. 3c (*left*) and Fig. 4 (*center* and *right*). *Right* and *dark green* represents reversal and curve, respectively. From **b** to **d**, acidic pH gradient used for simulations was as same as that shown in Fig. 3a, *left* to *right*. Conditions for (**d**) were used to obtain an optimal set of parameters. Parameters used were *α*_*c*_ 0.5, *β*_*c*_ 4.3, *α*_*r*_ 0.5, *β*_*r*_ *β*_*c*_ − 1.6, *φ*_*c*_ 30, *Δφ*_*c*_ 10, initial position of the model worm: <−2.0 (see Methods in detail). The ratio of reversal had a positive correlation with the angle in all conditions (R > 0.9, *p* < 0.01)

To evaluate the validity of the two-distinct-threshold model for navigational choice in acidic pH avoidance behavior in *C. elegans*, we conducted a computer simulation study by constructing a stochastic model for the behavior. As described above, we hypothesized that the decision between curve and reversal was driven by the absolute pH value detected by the worm. Therefore, in this model, we defined the probability of reorientation by two stochastic functions for curve and reversal. These functions were pH-dependent and each had a distinct threshold pH. Behavioral parameters of the model worm, such as speed [5] and degree of reorientation after reversal [21] were defined based on observations of the real worm. Parameters used in these functions were explored to reproduce the experimental data of real worms under the condition shown in Fig. 4d. Accordingly, we developed a model that had an apparent angle-dependent choice of behavioral maneuvers. The model was run on different conditions having acidic pH gradients steeper than a gradient used for model construction. The model could reproduce results similar to those observed in the real worm under steeper conditions (Fig. 5b, c; Additional file 3: Fig. S5), demonstrating the wide applicability of this model. Although the mean distances did not exactly replicate those of real worms, the angle distribution and shape of histogram resembled those of the real worm. These results strongly support the idea that worms choose a behavioral maneuver according to the distinct threshold pH value during avoidance behavior.

### Roles of glutamatergic neurotransmission on navigational choice

At least four classes of bilaterally symmetric chemosensory neuron pairs, ASE, ADF, ASH, and ASK are involved in acidic pH avoidance behavior in *C. elegans* [20]. Of these, ASH and ASK neurons are known to be glutamatergic because *eat*-*4*, a neuronal vesicular glutamate transporter gene necessary for glutamatergic neurotransmission, expressed in these neurons [22]. To study the role of glutamatergic signaling in the acidic pH avoidance behavior, the *eat*-*4(ky5)* mutant was used for behavioral analysis.

**Fig. 6:**
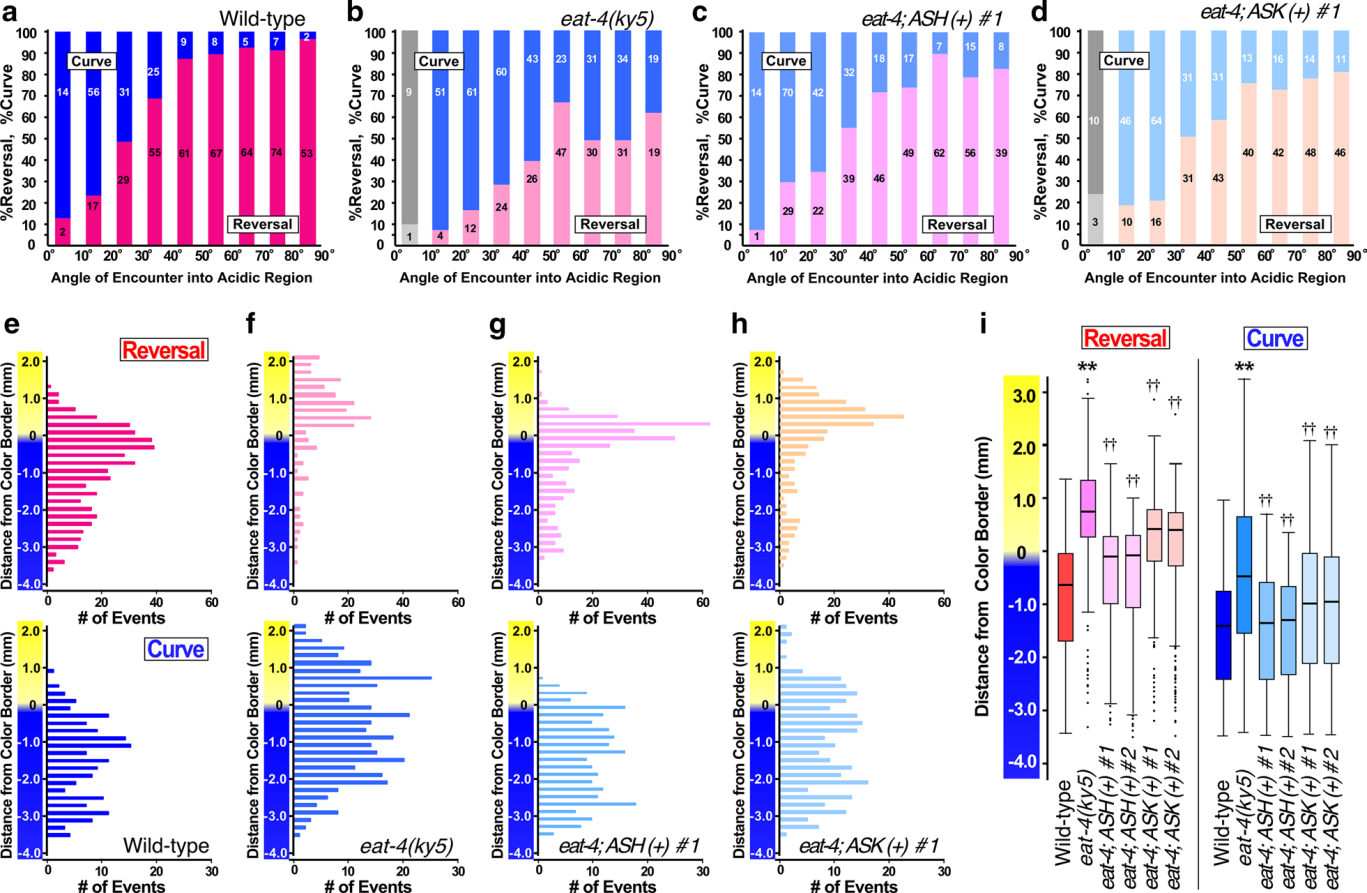
Acidic pH avoidance behavior of the *eat*-*4(ky5)* mutant. **a–d** Ratio of the behavioral choice, **e-h** histogram of distance and **i** boxplot of the distance distributions. Experiments were performed under the same conditions as in Fig. 2. Wild-type (**a**, **e**), *eat*-*4(ky5)* (**b**, **f**), *eat*-*4(ky5); ASH::eat*-*4(wt)* strain [ASH(+); **c**, **g**]. *eat*-*4(ky5); ASK::eat*-*4(wt)* [ASK(+); **d**, **h**] were examined. ASH (+) and ASK(+) are transgenic strains harboring a transgene driving ASH- and ASK-specific expression of the wild-type *eat*-*4* gene, respectively. The ratio of reversal had a positive correlation with the angle in all strains (R > 0.9, *p* < 0.01). In (**i**), statistical differences were examined using Student’s *t* test. *Asterisk* indicates a significant difference between wild-type and *eat*-*4(ky5)* (**p < 0.01). *Dagger* indicates a significant difference between the *eat*-*4(ky5)* and ASH(+), ASK(+) strain (^††^ *p* < 0.01)

Consistent with the notion that the *eat*-*4(ky5)* mutant has a defect in spontaneous backward locomotion upon food removal [23], the frequency of reversal avoidance was obviously reduced in the mutant (Fig. 6a, b, e, f). Moreover, the mean distance at which the reversal and curve avoidance occurred was significantly shifted to the acidic region (Fig. 6f, i). These results indicate that glutamatergic signaling in the *C. elegans* nervous system affects several aspects of acidic pH avoidance behavior including reversal frequency and worm position relative to the acidic color border, which reflects putative threshold pH for avoidance. Both reversal frequency and mean distance were partially recovered by ASH- and ASK-specific expression of wild-type *eat*-*4* cDNA (Fig. 6b–d, g–i) driven by *sra*-*6* and *sra*-*9* promoter, respectively [24]. Since many other glutamatergic neurons, such as interneurons, might be involved in the avoidance behavior, the sensory neuron specific expression was not sufficient to recover the phenotypic defects completely.

We next examined the acidic pH avoidance behavior of *rgs*-*3(vs19)*, *egl*-*3(n150)* and *egl*-*3(n729)*, to investigate the role of intensity of glutamatergic signaling from chemosensory neurons. The *rgs*-*3* gene encodes a *C. elegans* homolog of a mammalian RGS (regulator of G-protein signaling) proteins, and is expressed in a small subset of sensory neurons including ASH and ASK [25]. Loss of the *rgs*-*3* gene products suggested to cause a reduced glutamatergic output from the ASH synapse. In contrast, the *egl*-*3* mutant has enhanced glutamatergic output because the *egl*-*3* gene encodes an enzyme required for processing of the neuropeptide that downregulates glutamatergic output from ASH [26, 27].

**Fig. 7:**
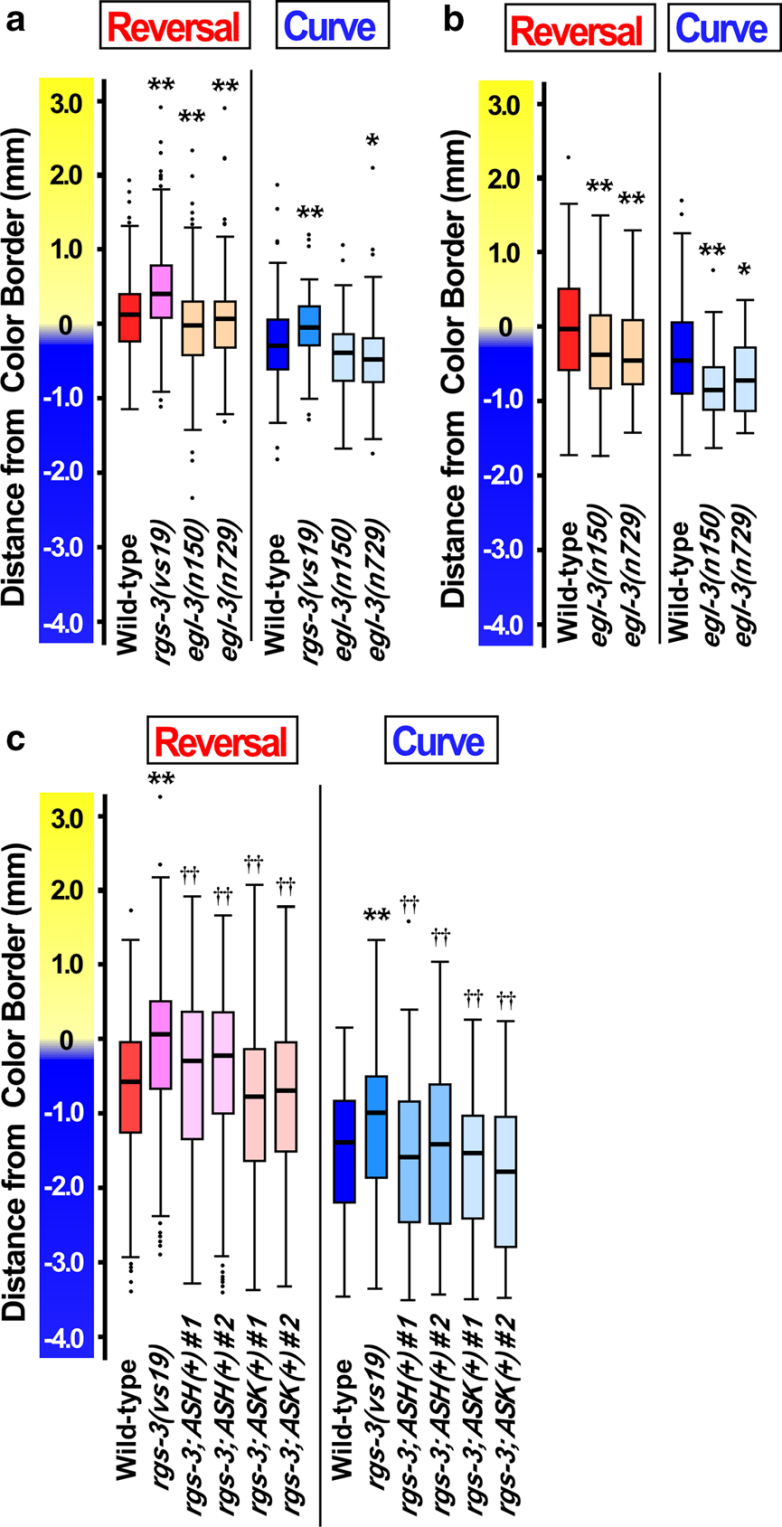
Acidic pH avoidance behavior of *rgs*-*3(vs19), egl*-*3(n159) and egl*-*3(n729)* mutants. **a** Boxplot of distance of reversal and curve avoidance relative to the acidic color border in glutamatergic mutants. The acidic pH gradient was formed using 30 mM HCl on 5 mM potassium phosphate buffer (see Additional file 3: Fig. S4 for steepness). Wild-type, *rgs*-*3(vs19), egl*-*3(n159), egl*-*3(n729)* strains were examined. **b** Boxplot of distance of reversal and curve avoidance of the *egl*-*3*mutants. The acidic pH gradient was formed using 10 mM HCl on 5 mM potassium phosphate buffer. Wild-type, *egl*-*3(n159), egl*-*3(n729)* strains were examined. **c** Boxplot of distance of reversal and curve avoidance relative to the acidic color border in *rgs*-*3(vs19)* mutants. The acidic pH gradient was formed using 30 mM HCl on 5 mM potassium phosphate buffer. Wild-type, *rgs*-*3(vs19), rgs*-*3(vs19); ASH::rgs*-*3(wt)* [ASH(+)], and *rgs*-*3(vs19); ASK::rgs*-*3(wt)* [ASK(+)] strains were examined. ASH(+) and ASK(+) are transgenic strains harboring a transgene driving ASH- and ASK-specific expression of the wild-type *rgs*-*3* gene, respectively. Statistical significances of difference were examined using Student’s *t*-test. *Asterisk* indicates a significant difference between wild-type and mutants (*p < 0.05, **p < 0.01). *Dagger* indicates a significant difference between the *rgs*-*3(vs19)* and ASH(+), ASK(+) strain (^††^ *p* < 0.01)

The apparent angle dependence of the navigational choice was not affected in *rgs*-*3(vs19)*, *egl*-*3(n150)*, and *egl*-*3(n729)* mutants (data not shown). However, the positions at which avoidance was executed were significantly different. In the *rgs*-*3(vs19)* mutant having reduced glutamatergic signaling, both reversal and curve avoidance occurred at more acidic regions than those in wild-type. Whereas in the *egl*-*3* mutants having enhanced glutamatergic signaling, both reversal and curve avoidance occurred at less acidic regions than wild-type (Fig. 7a). To further confirm the phenotype, the *egl*-*3* mutants were examined using slightly shallower gradient, and showed statistically significant differences from wild-type (Fig. 7b). Similar to the case for *eat*-*4* mutant, ASH- and ASK-specific expression of wild-type *rgs*-*3* recovered the phenotype (Fig. 7c). These results indicate that the glutamatergic signal output from ASH and ASK is involved in the regulation of both reversal and curve avoidance. Although the two threshold pHs are distinct, the neurotransmitter regulating these two behavioral maneuvers is not distinct. Large amounts of glutamate may be released from ASH and ASK in the presence of an intense stimulus, and small amounts of the transmitter may be released upon weak stimulation. Information about the ambient pH may be translated into the amount of glutamate synaptically released. In the *rgs*-*3* mutant having reduced glutamatergic output, ASH and ASK neurons require more intense stimulation to release the threshold amount of glutamate. A weaker stimulus is sufficient for ASH and ASK neurons in the *egl*-*3* mutant having enhanced glutamatergic output to reach the threshold.

## Discussion

### *C. elegans* appears to have two distinct threshold pHs for curve and reversal during acidic pH avoidance behavior

The locomotory sequences observed during acidic pH avoidance were quite similar to those observed in attraction behavior [5–10], behavior upon food removal [21, 23, 28, 29] and spontaneous locomotion [1, 2], suggesting that the avoidance behavior consists of intrinsic locomotory programs similar to other types of behavior in *C. elegans*.

**Fig. 8:**
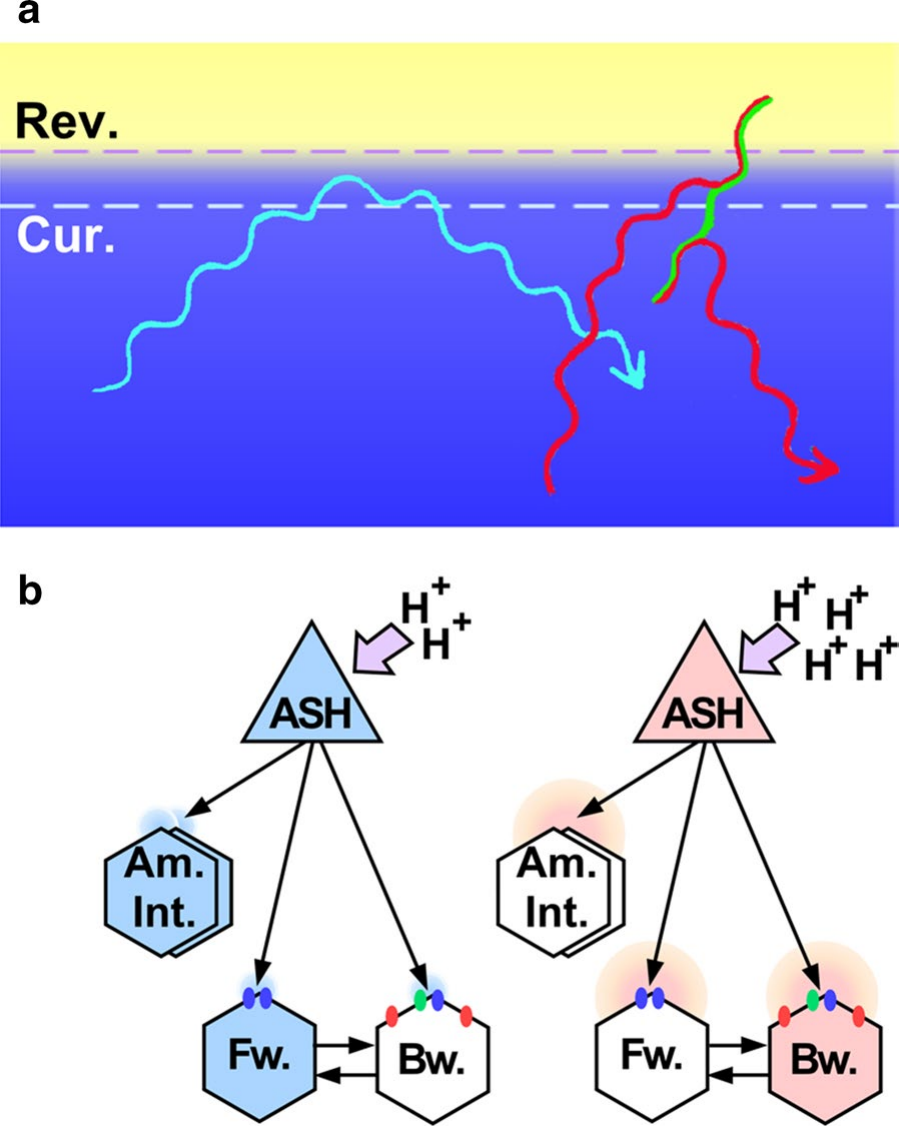
Schematic drawing of traces during acidic pH avoidance behavior of *C. elegans*. **a** The positions of the putative threshold pH for curve (Cur.) and reversal (Rev.) were indicated by a *broken line*. A *light blue* *line* represents a trace of forward locomotion during curve avoidance. *Red* and *green lines* represent traces of forward and backward locomotion during reversal avoidance, respectively. **b** Application of the differential activation model [28] on acidic pH avoidance behavior. Sensory neurons and interneurons were represented by triangle and hexagons, respectively. Synaptic connections were represented by *arrows*. Amphid interneurons and forward and backward command interneurons have been abbreviated as Am. Int., Fw., and Bw., respectively. *Blue and green dots* on forward and backward command interneurons represent synaptic GLR and GLC glutamate receptors, respectively. *Red dots* on backward command interneurons represent perisynaptic NMR receptors

In the positive chemotaxis and thermotaxis, behavioral strategies such as biased random walk and non-random steering are largely regulated by temporal variations in attractant concentration and ambient temperature caused by the worm locomotion [5, 6, 8, 13, 15, 16, 30]. Here, we propose that in the acidic pH avoidance behavior, both reversal (a form of biased random walk) and curve avoidance (a form of non-random steering) are regulated by a respective threshold pH. Wild-type worms initially executed curve avoidance at the relatively less acidic threshold pH for curve. If reorientation by curve avoidance was not sufficient and the worms reached the more acidic second threshold pH for reversal before they finished curving, they executed reversal (Fig. 8a). Although the behavioral maneuvers that occurred during the avoidance behavior were not completely sequential, and the worm positions at which they avoided the acidic pH were variable possibly because of the stochastic nature of behavioral decision making within the nervous system of the worm, the threshold model is plausible because in a population assay of the acidic pH avoidance behavior on a radial spatial gradient, the worm traces draw a definitive clear zone implying the threshold [20]. Furthermore, our computer simulation study substantiates the effectiveness of the two-threshold method during acidic pH avoidance behavior.

Although temporal variation in acidity seems to have no effect on the navigational choice of behavioral maneuver, temporal variation may play a role during curve avoidance. Forward locomotion of the worms after curve avoidance is biased in a less acidic direction. Worms may accomplish this non-random steering by detecting the variation in acidity with head swings, similar to those observed in isothermal tracking behavior and salt chemotaxis [8, 31]. Temporal variations may also be important when the worm is in the middle of an acidic pH gradient lower than pH 4.0. The threshold method is only effective when close to the threshold range.

The threshold method may be shared in avoidance behavior that has obvious thresholds in population assays, such as heavy metals [32] and osmotic avoidance [33]. This method, however, may not be common in all avoidance behavior. Iino and Yoshida [9] described that during sodium chloride avoidance after salt chemotaxis learning [34–36], the pirouette and weathervane responses are reversed. Worms move down the salt gradient as far as possible according to the temporal variation in the sodium chloride concentration.

In an attraction behavior, information about decrements in attractant concentration is as valuable as that about increments in the concentration. Whereas in acidic pH avoidance behavior, information about decreased acidity is not important for worms. At a pH 5.0–6.0, worms show no behavioral responses to ambient pH [20]. This disproportionate importance of information may play a role in the evolution of differences in behavioral regulation (threshold vs. temporal variation).

### Decoding of the threshold pH within a neuronal circuit of the worm

Our mutant analysis suggested that both reversal and curve avoidance are regulated, at least in part, by glutamatergic synaptic output from ASH and ASK neurons. As the intensity of the stimulus increases, the amount of glutamate released from these neurons to synaptic clefts may also increase. Neurons postsynaptic to these neurons may decode the information about the intensity of signals by referring an intrinsically encoded threshold amount of glutamate. Backward locomotion is promoted during reversal avoidance, while forward locomotion is promoted during curve avoidance. How does a single neurotransmitter released from a small number of sensory neurons regulate two mutually exclusive behavioral responses? An important insight was suggested by Mellem et al. [27]. Based on their integrative study using genetics and electrophysiology, they proposed a “differential activation model” in which a specific level of glutamate released from ASH causes a distinct response in the postsynaptic neurons. In their model, a small amount of glutamate activates GLRs (and possibly GLC) synaptically-localized and a large amount of glutamate activates perisynaptic NMR receptors in addition to GLR/GLC at the synapse between ASH and backward command interneurons. According to the wiring diagram of the worm neuronal network drawn by White et al. [37], a bilateral pair of ASH neurons sends their synaptic outputs to the backward command interneurons AVA, AVD, and AVE as well as to the forward command interneuron AVB. In acidic pH avoidance behavior, a small amount of glutamate released by ASH may activate synaptic GLR receptors on both backward and forward command interneurons. In addition, GLC receptors may also be activated in ASH-backward interneuron synapses, thus downregulating neuronal excitation. Overall, forward locomotion is promoted by weaker stimulation during curve avoidance. In this situation, amphid interneurons downstream to ASH and/or ASK may be activated for regulation of non-random steering. In the presence of an intense stimulus, backward command interneurons may be more activated through perisynaptic NMR receptors by glutamate spilled out from the synapse, thereby promoting backward locomotion (Fig. 8b, Additional file 3: Fig. S6). Although the role of ASK is not clear, ASK may contribute to the regulation of reversal avoidance via pathway yet unidentified, because ASK has an important role on promoting backward locomotion [29].

Cell ablation studies on both sensory and interneurons, analysis of mutants defective in the postsynaptic glutamate receptors, physiological analysis either by electrophysiological or optical methods may help us understanding fully the mechanism of behavioral regulation during the acidic pH avoidance behavior. In this context, it should be noted that the acid-sensing chemosensory neurons ASE and ADF send their synaptic outputs mainly to AIY and AIZ interneurons, respectively, which may be involved in the regulation of non-random steering [9].

## Conclusions

*C. elegans* avoids acidic pH using two behavioral maneuvers, reversal (klinokinesis) and curve (klinotaxis). It is widely accepted that in positive chemotaxis, *C. elegans* detects temporal derivative of stimulus concentration (*dC/dt*) to regulate reversal locomotion. However, in acidic pH avoidance, probability of reversal and curve avoidances were dependent on absolute pH rather than *dC*/*dt*. The pH threshold is different between reversal and curve avoidance. Curve avoidance always occurred at less acidic region than reversal, irrespective of steepness of the acidic pH gradient. Mutant studies suggested that the difference in threshold pH results from a differential amount of glutamate released from ASH and ASK chemosensory neurons in response to stimulus intensity.

## Methods

### Strains and culture

The wild-type animal used in this study was *Caenorhabditis elegans ver*. Bristol, strain N2. Worms were grown under standard conditions at 20 °C [38]. Mutant strains used were CX5 *eat*-*4(ky5)* III, LX242 *rgs*-*3(vs19)* II, MT1541 *egl*-*3(n729)* V, MT150 *egl*-*3(n150)* V, obtained from Caenorhabditis Genetics Center (CGC). The transgenic strains used were WKB70 *rgs*-*3(vs19)* II; *iwtEx50[sra*-*6::rgs*-*3cDNA; myo*-*3::RFP]*, WKB71 *rgs*-*3(vs19)* II; *iwtEx51[sra*-*6::rgs*-*3cDNA; myo*-*3::RFP]*, WKB72 *rgs*-*3(vs19) II; iwtEx52[sra*-*9::rgs*-*3cDNA; unc*-*122::mCherry]*, WKB73 *rgs*-*3(vs19) II; iwtEx53[sra*-*9::rgs*-*3cDNA; unc*-*122::mCherry]*,WKB77 *eat*-*4(ky5)III; iwtEx57[sra*-*9::eat*-*4cDNA, mec*-*4::GFP]*, WKB78 *eat*-*4(ky5)III; iwtEx58[sra*-*9::eat*-*4cDNA, mec*-*4::GFP]*, WKB79 *eat*-*4(ky5)III; iwtEx59[sra*-*6::eat*-*4cDNA, mec*-*4::GFP]*, WKB80 *eat*-*4(ky5)III; iwtEx60[sra*-*6::eat*-*4cDNA, mec*-*4::GFP]*. We used *sra*-*6* and *sra*-*9* promoters for cell-specific expression of cDNA in ASH and ASK neurons, respectively [24]. Germline transformation of mutants were performed by microinjecting a mixture of rescue construct (20 ng/μl) and marker construct (80 ng/μl) into hermaphrodite gonad according to Mello et al. [39].

### Analysis of acidic pH avoidance behavior

A 9**-**cm petri dish containing 10 ml of 2 % agar, 5 or 0.2 mM sodium acetate pH 6.0 (pH adjusted with acetic acid), 50 mM NaCl, 1 mM CaCl_2_, and 1 mM MgSO_4_ was used as an assay plate. In some experiments, 5 mM potassium phosphate (pH 6.0) was also used instead of sodium acetate. To visualize acidic regions on the plate, 1 ml of water-saturated bromophenol blue (BPB) solution was included in the gel. To form the acidic pH gradient, 2 % acidic agar gels containing 100 mM or 30 mM HCl were prepared by mixing equal volumes of 4 % agar solution and 200 mM or 60 mM HCl solution maintained at 60 °C, then pouring into a rectangular plastic dish (W 8 cm × L 12 cm). Three to four strips of the acidic agar gel (W 5 mm × L 45 mm × H 1 mm) were excised from the dish, arranged in a triangle or rectangle on the assay plate. The assay plate with acidic agar gel strips was left for 30 min at 20 °C until an acidic pH gradient formed (Fig. 1a). Although the acidic yellow region was expanded over time, relationship between yellow color border and worm avoidance behaviors (distance and angle, see below) were not changed with the time.

A single well-fed young adult *C. elegans* was transferred to the center of the assay plate, and allowed to move freely on the plate for 50 min. The behavior of the worm was tracked using automatic tracking system [40] and was recorded. Movies were analyzed using custom-made software. Acidic pH avoidance behavior by reversal or curve were identified by watching the movies and were recorded along with the time, angle of encounter to the acidic yellow border, and the distance of the worm position relative to the yellow border when the worm moved furthest into the acidic region during the avoidance behavior (Fig. 1b–e). Reversal locomotion of three or more head swings were referred to long and that of two or less head swings were referred to short reversals, deep body bendings (omega-shaped turns) were visually identified by the head nearly touching the tail as described by Gray et al. [21]. Gradual curves were also visually identified when worms change their direction less than 90° within single bend as described by Kim et al. [2]. Although the wild-type *C. elegans* consistently avoided the acidic region, it occasionally failed in the process of achieving eventual avoidance. In this situation, the worm showed multiple consecutive avoidance behavior, until it succeeded in avoiding the acidic region. We only analyzed the first occurrence of the serial avoidance to precisely evaluate the relationship between the initial choice of behavioral maneuvers and the two parameters.

### pH measurements

Six thin (1 mm in width) strips of agar gel around the acidic yellow border were excised from the assay plates (−3 to +3 mm, as indicated in Fig. 1f) and collected individually at the bottom of separate 1.5 ml sample tubes. The pH of each of the gel strips was measured by a micro pH electrode (9669-10D, HORIBA, Tokyo, Japan).

### Computer simulation

In this model, the location of the worms were represented as a point (*x*_*t*_, *y*_*t*_) almost corresponding to their center of mass (Fig. 5a). The model worms moved on a virtual field that had an acidic pH gradient, same as that shown in Fig. 3a. The model worms moved in a straight line in one direction at a constant speed (0.15 mm/s), updating their position and direction every 0.8 s. The model worms had a pH sensor 0.51 mm ahead of its positional point and swung it side by side, as shown in Fig. 5a. The sensor detected the ambient pH every 0.8 s. The initial position of the model worm was randomly determined within the less acidic region of the virtual field (Additional file 3: Fig. S5A). To represent the frequency of the angle of encounter in the real worm, the initial direction *θ*_*0*_ (−90° < *θ*_*0*_ ≤ 90°) of the worm movement was selected from random values having 1 − |*sinθ|* distribution.

The model worm implemented two types of reorientation mechanisms corresponding to curve and reversal. Both reorientation events occurred stochastically, depending on the pH value detected. The probability of curve was defined by the following equation:

1 $${p_{c} \left( C \right) = \frac{ 1}{{ 1+ { \exp }\left( {\frac{{C - \beta_{c} }}{{\alpha_{c} }}} \right)}}}$$

Similarly, the probability of reversal was defined by the equation:

2 $${p_{r} \left( C \right) = \frac{ 1}{{ 1+ { \exp }\left( {\frac{{C - \beta_{r} }}{{\alpha_{r} }}} \right)}}}$$

where *C* is a pH value detected by the model worm, *α*_*c*_ and *β*_*c*_ correspond to deviation and center of probability density function for curve, respectively, and *α*_*r*_ and *β*_*r*_ correspond to those for reversal, respectively. These parameters conferred threshold pH and variation for each reorientation on the model worm. The model worm reoriented in a new direction when the curve or reversal was determined. If the curve and reversal were determined at the same time point, reversal was selected. The degree of reorientation in reversal was chosen from a normally distributed random value having 137° ± 8° distributions [25]. The direction of reorientation in reversal was determined randomly toward either side. The magnitude of reorientation in curve was chosen from a normally distributed random value having *φ*_*c*_ ± *Δφ*_*c*_ distribution, in which *φ*_*c*_ and *Δφ*_*c*_ were determined by parameter optimization (see below). The direction of reorientation in curve, determined at time *t*_*n*_, was selected by comparing the pH detected at *t*_*n*_ and *t*_*n*−*1*_. The model worm reoriented toward the less acidic side. The behavior of the model worms started at time *t*_*0*_ and ended when the worms reoriented in a less acidic direction by either of the two reorientation maneuvers. The type of reorientation maneuver selected at the end of the behavior was recorded along with the position of the model worm and direction of movement immediately before the reorientation.

Parameter sets (*α*_*c*_, *β*_*c*_, *α*_*r*_, *β*_*r*_ in pH values) in Eqs. (1) and (2) and degrees of curve reorientations (*φ*_*c*_ ± *Δφ*_*c*_ in angle) were selected from several values. Parameter values used were *α*_*c*_ (0.125, 0.25, 0.5), *β*_*c*_ (2.8, 3.3, 3.8, 4.3), *α*_*r*_ (0.125, 0.25, 0.5), *β*_*r*_ (*β*_*c*_ − 0.4, *β*_*c*_ − 0.8, *β*_*c*_ − 01.2, *β*_*c*_ − 1.6), *φ*_*c*_ (30, 60, 80), *Δφ*_*c*_ (10, 30, 60), and initial position of the model worm (<−3.0, <−2.0, <−1.0 in mm). Simulations were performed on 3000 model worms for every possible combination of parameter sets (2916 combinations). A set of parameters that produced results matching the experimental data of the real worm shown in Fig. 4d were selected (Fig. 5d). The same parameter set was used for analysis under different conditions with steeper pH gradients (Fig. 5b, c; Additional file 3: Fig S5A–C).

All programs were written from scratch by using C and Ruby.

## Availability of supporting data

The source code for computer simulation study is available from https://github.com/kzmsakata/threshold_turn.

## Additional files

10.1186/s12868-015-0220-0 A movie for long reversal avoidance of wild-type *C. elegans*. The bright region on the right shows the acidic yellow region and the dark region shows the less acidic region.
10.1186/s12868-015-0220-0 A movie for gradual curve avoidance of wild-type *C. elegans*. The bright region on the left shows acidic yellow region and the dark region shows the less acidic region.
10.1186/s12868-015-0220-0 Supplemental Figures S3–S6.

## Abbreviation

BPB: bromophenol blue
