# Supplementary material for: Navigational choice between reversal and curve during acidic pH avoidance behavior in Caenorhabditis elegans
Source: BMC Neurosci. 2015 Nov 19;16:79. doi: 10.1186/s12868-015-0220-0 (PMC4653917; doi:10.1186/s12868-015-0220-0)
Supplement: Supplementary file 3 — 10.1186/s12868-015-0220-0 Supplemental Figures S3–S6. [file 12868_2015_220_MOESM3_ESM.docx]

Additional Figures for:

**Navigational choice between reversal and curve during acidic pH avoidance behavior in *Caenorhabditis elegans*.**

Wakabayashi, T and Sakata, K *et al.*

**
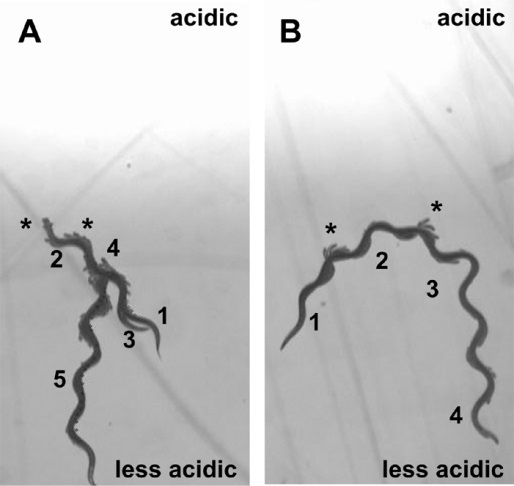
Fig. S3** Overlaid images of long reversal avoidance **(A)** and gradual curve avoidance **(B)** from Supplemental Movies S1 and S2. In **(A)**, (1) forward locomotion toward the acidic region, (2) stop, (3) backward, (4) turn by deep body bending, and (5) forward locomotion in the less acidic direction are indicated. In **(B)**, (1) forward locomotion toward the acidic region, (2) (3) gradual turns by asymmetric body bending, and (4) forward locomotion in the less acidic direction are indicated. Small head swings similar to the stomatal oscillations described by Croll [1] were indicated by asterisks. The bright region represents the acidic yellow region.

**
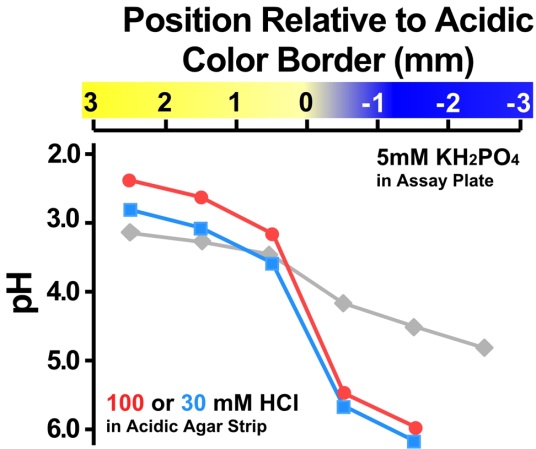
Fig. S4** Steepness of the acidic pH gradient used for the experiments in Fig.2, 6, and 7, formed using acidic agar strips containing 100 mM (*red*), 30 mM (*blue*) of HCl on an assay plate containing 5 mM potassium phosphate buffer. For comparison, a shallow gradient using 30 mM HCl for gradient on 0.2 mM sodium acetate buffer is also shown (*Gray*).

**Fig. S5** **(A)** Initial position of the model worms. The white arrow indicates the initial position and direction of the model worm on the virtual field having an acidic pH gradient. **(B)** Traces of model worms avoided the acidic pH by reversal. Twenty randomly selected traces before (20 steps) and after (two steps) reversal are shown. Traces immediately before reversal avoidance (five steps) aligned at the position of reorientation are shown in an inset (scale bar 0.2 mm). **(C)** Traces of model worms avoided the acidic pH by curve. Twenty randomly selected traces before (20 steps) and after (two steps) curve were shown. Traces immediately before curve avoidance (five steps) aligned at the position of reorientation are shown in an inset (scale bar 0.2 mm). Parameters used were as same as those in **Fig.5**.


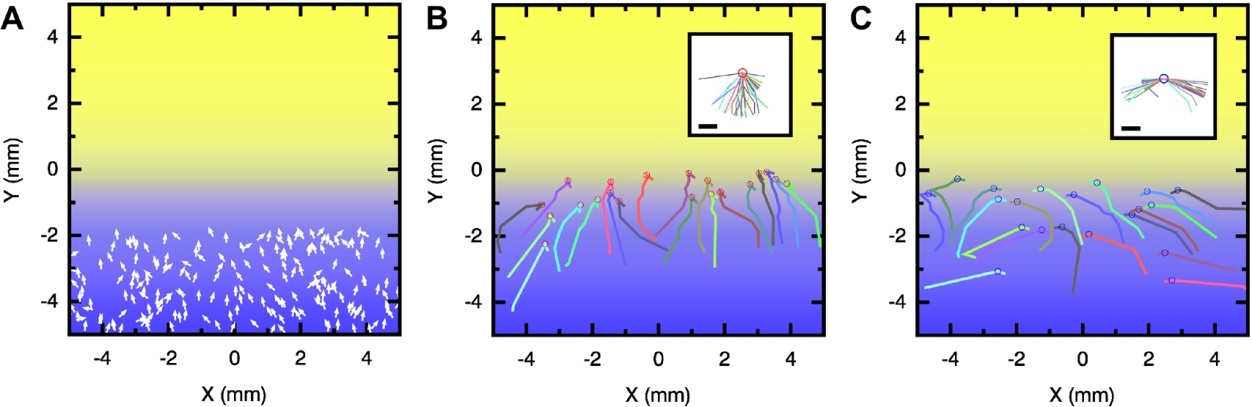


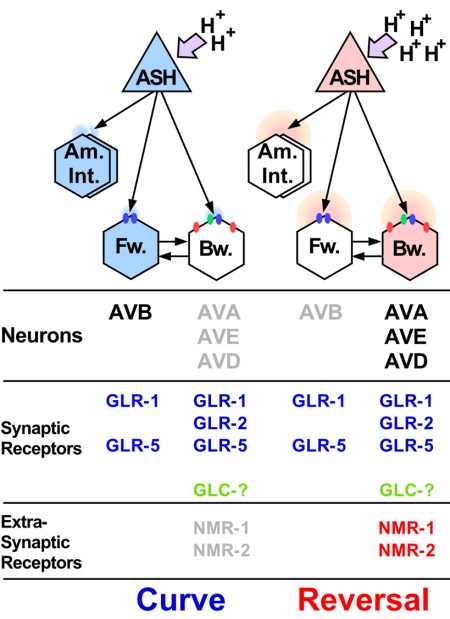
**Fig. S6** Application of the differential activation model on acidic pH avoidance behavior. Neurons are represented as in **Fig. 8B**. Neurons included in the forward and backward command interneurons, synaptic and extra-synaptic glutamate receptors expressed [28] [S1] [S2] have been indicated below. Distinct subsets of glutamate receptor genes are expressed in these forward/backward command interneurons [S1]. In the AVA, AVD, and AVE neurons, the non-NMDA glutamate receptors *glr-1*, *glr-2*, and *glr-5* and the NMDA receptors *nmr-1*, and *nmr-2* are expressed. Although the gene identity was not determined, electrophysiological analysis suggests that glutamate-gated chloride channel gene(s) (*glc*) are also expressed in AVA neurons [28]. On the other hand, AVB interneurons express *glr-1* and *glr-5*. The GLR-1 and GLR-2 proteins are co-localized at the synapse [28], while NMR-1 proteins are distributed at extrasynaptic sites of backward command interneurons AVA, AVD, and AVE [S2]. Neurons and receptors that may be activated during curve (left) and reversal (right) avoidances have been highlighted.

**Additional Reference**

S1. Brockie PJ, Madsen DM, Zheng Y, Mellem J, Maricq AV: [**Differential expression of glutamate receptor subunits in the nervous system of *Caenorhabditis elegans* and their regulation by the homeodomain protein UNC-42.**](http://www.ncbi.nlm.nih.gov/pubmed/11222641) *J Neurosci* 2001a, **21:** 1510-1522.

S2. Brockie PJ, Mellem JE, Hills T, Madsen DM, Maricq AV: **The *C.* *elegans* glutamate receptor subunit NMR-1 is required for slow NMDA-activated currents that regulate reversal frequency during locomotion.** *Neuron* 2001b, **31:** 617-630.
